# Effective Management of Sacral Stress Fractures in Gastric Cancer: Iliosacral Screw Fixation Following a Type 3 Hemipelvectomy

**DOI:** 10.7759/cureus.56435

**Published:** 2024-03-19

**Authors:** Takaki Kitamura, Tomonori Shigemura, Yohei Yamamoto, Yasuaki Murata

**Affiliations:** 1 Department of Orthopaedic Surgery, Graduate School of Medicine, Chiba University, Chiba, JPN; 2 Department of Orthopaedics, Teikyo University Chiba Medical Center, Ichihara, JPN

**Keywords:** oncologic orthopedics, iliosacral screw fixation, type 3 internal hemipelvectomy, sacral stress fracture, metastatic pelvic tumors

## Abstract

Metastatic pelvic tumors pose a significant challenge in oncologic orthopedics due to their complex management and the high potential for postoperative complications. This case study discusses a 75-year-old male with a sacral stress fracture following a type 3 internal hemipelvectomy for a metastatic lesion from gastric cancer in the left pubic bone. Initial conservative treatments failed to yield satisfactory improvement, leading to surgical intervention. Open reduction and internal fixation with an iliosacral screw, despite complications, significantly alleviated pain and improved mobility. This case underscores the difficulty in diagnosing sacral stress fractures versus metastatic lesions and highlights the effectiveness of iliosacral screw fixation in managing postoperative sacral stress fractures. It emphasizes the procedure’s role in providing early pain relief and enhancing daily activity levels. Additionally, it points out the importance of addressing altered bone metabolism in the postoperative care of patients with metastatic pelvic tumors. This contributes to the literature by stressing the incidence of sacral stress fractures as a critical, though often overlooked, complication and demonstrating the benefits of iliosacral screw fixation in such scenarios for better recovery and quality of life.

## Introduction

Metastatic pelvic tumors, often secondary to primary malignancies, represent a formidable challenge in oncologic orthopedics [1]. These tumors commonly metastasize to the pelvis, contributing to significant morbidity due to their propensity to cause pain, structural instability, and functional impairment [2]. The treatment paradigm for these lesions has traditionally spanned from conservative management to more radical interventions like hemipelvectomy, a procedure necessitated by the extent of tumor invasion and associated complications [3]. Despite advances in surgical techniques, the management of postoperative complications, particularly sacral stress fractures, remains a clinical enigma [4]. These fractures, while recognized as a potential complication, are frequently underdiagnosed due to their subtle presentation, thus complicating the postoperative recovery in patients already burdened with malignancy.

In this case study, we detail the complex management of a sacral stress fracture that occurred after the resection of pubic rami due to a solitary metastasis in the left pubic area from gastric cancer. The case posed a perplexing problem, involving the need to distinguish the fracture from its pathological counterpart and requiring careful surgical treatment through open reduction and internal fixation (ORIF) using an iliosacral screw. This method not only significantly reduced the patient's pain from the initial stages but also enabled the patient to walk independently.

## Case presentation

**Figure 1:**
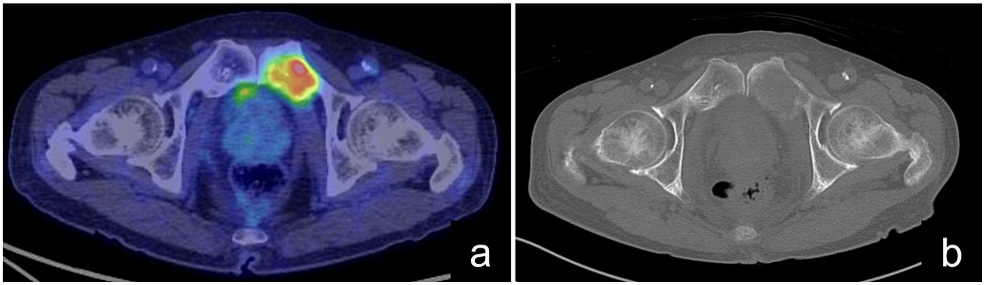
Metastatic pelvic tumor in the left pubic bone. (a) Positron emission tomography-computed tomography revealed anomalous accumulation in the left pubic bone. (b) Computed tomography imaging detected myocytic alterations in the left pubic bone.

A 75-year-old man, previously subjected to laparoscopic distal gastrectomy for gastric cancer, presented with a complex clinical scenario. Six years following his initial surgery, a positron emission tomography-computed tomography (PET-CT) scan revealed a single hyperaccumulation in the left pubic bone, indicative of a metastatic spread, yet the patient exhibited no symptomatic manifestations at this stage (Figure 1).

**Figure 2:**
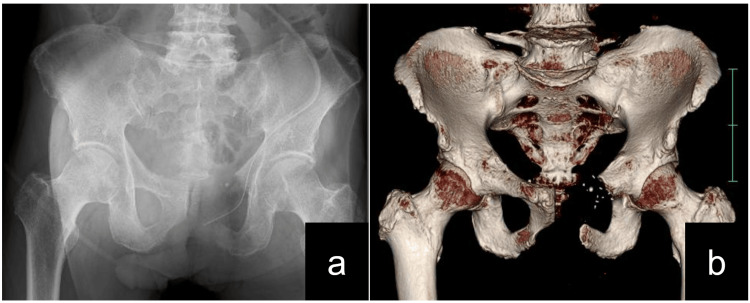
After a type 3 internal hemipelvectomy. (a) Postsurgical radiograph following a type 3 internal hemipelvectomy. (b) Three-dimensional computed tomography postoperative image after a type 3 internal hemipelvectomy.

After the PET-CT scan, a biopsy confirmed gastric cancer metastasis to the pubic bone. Prognostic evaluations, as indicated by a Katagiri score of 5 [5], suggested a life expectancy extending beyond six months. Given the solitary nature of the metastasis, he was deemed a suitable candidate for surgical intervention, leading to the performance of a type 3 internal hemipelvectomy at a specialized center (Figure 2) [6].

This procedure, however, was complicated by extraosseous invasion and a positive surgical margin, necessitating further postoperative radiotherapy.

**Figure 3:**
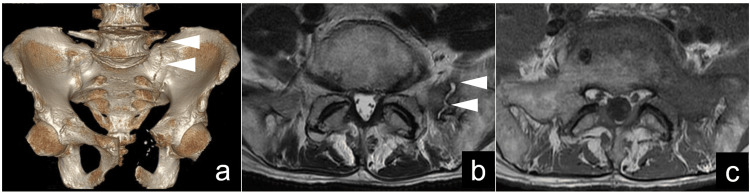
A fracture line in the left sacrum. (a) Three-dimensional computed tomography discloses a fracture line within the left sacrum. (b) T2-weighted magnetic resonance imaging demonstrates heightened signal intensity in the left sacrum. (c) T1-weighted magnetic Resonance imaging exhibits diminished signal intensity in the left sacrum.

In the postoperative phase, the patient engaged in an unrestricted rehabilitation regimen, maintaining an initial performance status of 1. However, approximately one month after the tumor removal, he developed severe pain in the lower left back, accompanied by a significant decline in mobility without any trauma. Diagnostic imaging, including CT and magnetic resonance imaging (MRI), unveiled a fracture line in the left sacrum, along with a corresponding low-signal area on T1-weighted images and high-signal area on T2-weighted images (Figure 3).

Laboratory tests revealed a low-serum 25(OH) vitamin D level of 12.4 ng/mL, while calcium, phosphorus, and fibroblast growth factor 23 (FGF23) levels remained within normal limits. Bone mineral density (BMD) assessments using dual-energy X-ray absorptiometry reported a young adult mean above 80%. A subsequent bone biopsy ruled out a pathological fracture, confirming it as a sacral stress fracture after a type 3 internal hemipelvectomy with vitamin D deficiency due to postgastrectomy bone disorder. Initially opting for a conservative treatment approach with weight-bearing therapy and vitamin D administration, the patient experienced some improvement in pain, and CT scans showed evidence of callus formation. Consequently, a transition to partial weight-bearing was initiated. Nonetheless, this shift led to a recurrence of pain and further mobility challenges. The patient's performance status had deteriorated to 3.

**Figure 4:**
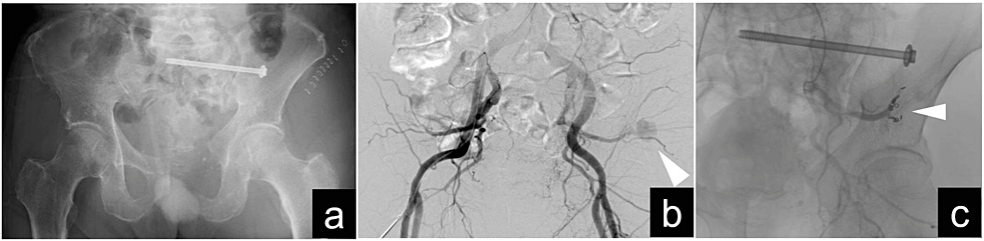
Postoperative radiograph and intravascular radiology imaging. (a) Anteroposterior radiograph after open reduction and internal fixation utilizing an iliosacral screw. (b) Intravascular radiology imaging captured hemorrhaging from the superior gluteal artery. (c) Intravascular radiology imaging confirmed coil embolization of the hemorrhagic site.

Given the patient's strong desire for ambulation and limited success with conservative therapy, a decision was made to pursue surgical treatment. Surgical methods can be categorized into two primary types: posterior pelvic fixation and lumbopelvic fixation techniques. Furthermore, these procedures can be further subdivided into percutaneous surgeries or open surgeries. In this case, given the patient's cancer status and the presence of a nondisplaced sacral fracture, a less invasive surgical option was chosen [7]. Four months after the initial hemipelvectomy, he underwent ORIF using an iliosacral screw. This procedure was not without complications; the insertion of the screw into S2 inadvertently injured the superior gluteal artery, necessitating additional hemostatic treatment through interventional radiology (Figure 4).

**Figure 5:**
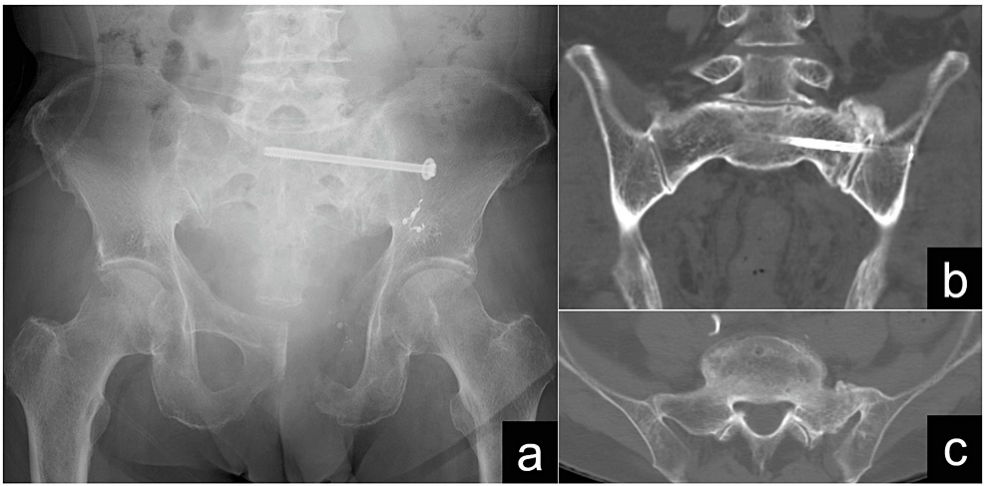
Twelve-week follow-up. (a) Anteroposterior radiograph 12 weeks after open reduction and internal fixation (ORIF). (b) Coronal computed tomography image illustrating cranial bone fusion at 12 weeks post-ORIF. (c) Axial computed tomography image depicting ventral bone fusion at 12 weeks post-ORIF.

Post-surgery, the patient embarked on an unrestricted walking training program, and within three weeks, he was discharged from the hospital, walking independently. Outpatient anticancer treatment was resumed eight weeks following the surgery. Bone union was confirmed via CT at the 12-week follow-up (Figure 5).

There were notable improvements in his pain Numerical Rating Scale and EQ-5D-5L scores over time (Table 1) [8].

**Table 1:** Changes in EQ-5D-5L, NRS, and PS over time. ^a^EQ-5D-5L is an index that assesses health status across five domains: mobility, self-care, usual activities, pain/discomfort, and anxiety/depression. Each domain has five response levels, with 1 indicating the best state and 5 indicating the worst state. It can represent a total of 3,125 different health states, ranging from 11,111 to 55,555. ^b^The Numerical Rating Scale (NRS) is a tool used to quantify the pain experienced by patients. This scale evaluates the intensity of pain on a scale of 0 to 10, across 11 levels. A score of 0 means *no pain*, while 10 represents *the worst pain imaginable*. ^c^The Performance Status (PS) scale assesses cancer patients' health, ranging from PS 0, indicating no restrictions and the best condition, to PS 4, denoting complete immobility and the worst state. This system aids in guiding cancer treatment decisions and evaluating patient risks.

	EQ-5D-5L^a^					NRS^b^	PS^c^
Time	Mobility	Self-care	Usual activities	Pain/discomfort	Anxiety/depression		
Preoperation	3	5	2	4	4	8	3
8 weeks after surgery	2	1	2	2	1	2	1
12 weeks after surgery	2	1	2	2	1	1	1

## Discussion

The clinical narrative of this case reveals two pivotal findings that enrich the current understanding of the management of metastatic pelvic tumors. First, our observations confirm the occurrence of sacral stress fractures as a notable postoperative complication following a type 3 internal hemipelvectomy. Second, the case effectively demonstrates the therapeutic value of ORIF using an iliosacral screw for managing sacral stress fractures post-hemipelvectomy. These outcomes suggest that iliosacral screw fixation could play a vital role in the postoperative management of similar complex cases.

Sacral stress fractures following a type 3 internal hemipelvectomy, as observed in metastatic pelvic tumors, present a unique clinical challenge. In cases like the one presented, where type 3 metastases are involved, postoperative activities of daily living (ADLs) are often better preserved compared to types 1 and 2 metastases [3]. However, the lack of bone reconstruction in these scenarios can lead to increased mechanical stress on the sacrum. In cancer patients, distinguishing fragile sacral fractures from pathological fractures due to metastatic bone tumors is crucial, often necessitating bone biopsy for accurate diagnosis [9]. This complexity can impede early detection. However, in patients undergoing post-type 3 hemipelvectomy, when fracture lines are clearly visible on MRI or CT scans, as in the present case, the likelihood of sacral insufficiency fractures is high. Therefore, a conservative observation approach may allow for the avoidance of biopsy. Moreover, as evidenced in this case, lower back pain could be an initial symptom, potentially leading to missed diagnoses if investigations such as MRI are limited to the lumbar spine alone. Therefore, in patients presenting with lower back pain post-hemipelvectomy, a high index of suspicion for sacral stress fractures is warranted.

ORIF using an iliosacral screw has emerged as a valuable treatment for sacral stress fractures resistant to conservative management, particularly following a type 3 internal hemipelvectomy in the context of metastatic pelvic tumors. The standard management of most pelvic tumors typically encompasses radiation therapy and analgesic measures. In cases where pain remains uncontrolled and structural instability persists, methods like local excision, cement augmentation, and regional reconstruction are often employed, especially in regions outside the acetabulum [3]. Recent advancements suggest the utility of percutaneous screw fixation for non-acetabular metastatic bone tumors and sacral fractures, especially in cases where pain management is challenging [10,11]. Resection is typically reserved for situations with a favorable prognosis, such as isolated metastasis or primary bone tumors, as exemplified in this case. Traditionally, a type 3 internal hemipelvectomy has been associated with positive outcomes, often not necessitating bony reconstruction due to the preservation of sufficient functional capacity through excision alone [3]. Previous literature documenting sacral fractures following a type 3 internal hemipelvectomy generally indicates successful bone union with conservative treatment modalities [4]. However, in the presented case, conservative management over three months postoperatively did not yield successful outcomes for the sacral stress fracture. A finite element analysis suggests that employing two transiliac-transsacral screws is the preferred method for posterior pelvic fixation [12]. Despite this, the surgical strategy was adapted to utilize an iliosacral screw at S1 and a transiliac-transsacral screw at S2, a decision influenced by the distinct shape of the right sacrum. However, complications related to arterial injury necessitated a modification of the plan, resulting in the implementation of a single-screw fixation at S1. Despite the alteration in surgical technique, this intervention allowed for the early alleviation of pain and the commencement of weight-bearing activities, significantly contributing to the recovery of the patient's ADL. Given that this case involved a sacral insufficiency fracture without displacement, the fixation with a single iliosacral screw may have been sufficient.

The etiology of sacral stress fractures following type 3 resection in patients with metastatic pelvic tumors could be attributed to altered bone metabolism secondary to gastrectomy. Gastrectomy, a common surgical intervention for gastric cancer, can have far-reaching effects on bone health, primarily due to the resultant malabsorption of essential nutrients vital for maintaining bone integrity [13,14]. This procedure often leads to deficiencies in vitamin D and calcium, key components in bone metabolism, thus predisposing patients to an increased risk of fractures. In the context of a type 3 internal hemipelvectomy, the structural alterations and biomechanical stresses imposed on the pelvic region further exacerbate this risk. The resection, while necessary for managing the tumor, disrupts the normal load distribution across the pelvis, subsequently placing undue stress on the sacrum. When coupled with weakened bone quality due to post-gastrectomy metabolic changes, this creates a conducive environment for the development of stress fractures. Therefore, it is imperative in such cases to consider and address the potential impact of gastrectomy on bone health as part of the comprehensive management plan for patients undergoing type 3 resections for metastatic pelvic tumors.

## Conclusions

In conclusion, this case study illuminates two key aspects of managing post-hemipelvectomy complications in patients with metastatic pelvic tumors: the critical need for vigilant monitoring for sacral stress fractures and the effectiveness of iliosacral screw fixation in addressing these fractures. The insights gleaned from this case are invaluable in enhancing postoperative care and could inform future clinical practices, potentially improving outcomes for patients undergoing similar treatments.

## References

[1] Yang Q, Chen N, Fu W (2021). Clinical features and outcomes of metastatic bone tumors of the pelvis. J Int Med Res.

[2] Chu EC, Lee WT (2023). Prostate cancer presenting as hip pain at the chiropractic office: a case report and literature review. Cureus.

[3] Mayerson JL, Wooldridge AN, Scharschmidt TJ (2014). Pelvic resection: current concepts. J Am Acad Orthop Surg.

[4] Imanishi J, Yazawa Y, Oda H, Okubo T (2015). Type 3 internal hemipelvectomy: a report of two cases. J Orthop Surg (Hong Kong).

[5] Katagiri H, Okada R, Takagi T (2014). New prognostic factors and scoring system for patients with skeletal metastasis. Cancer Med.

[6] Enneking WF, Dunham WK (1978). Resection and reconstruction for primary neoplasms involving the innominate bone. J Bone Joint Surg Am.

[7] Santolini E, Kanakaris NK, Giannoudis PV (2020). Sacral fractures: issues, challenges, solutions. EFORT Open Rev.

[8] Shiroiwa T, Ikeda S, Noto S, Igarashi A, Fukuda T, Saito S, Shimozuma K (2016). Comparison of value set based on DCE and/or tto data: scoring for EQ-5D-5L health states in Japan. Value Health.

[9] Donovan A, Schweitzer ME, Rafii M, Lax A (2009). Radiological features of superomedial iliac insufficiency fractures: a possible mimicker of metastatic disease. Skeletal Radiol.

[10] Yang R, Goch A, Murphy D (2020). A novel tripod percutaneous reconstruction technique in Periacetabular lesions caused by metastatic cancer. J Bone Joint Surg Am.

[11] Rommens PM, Wagner D, Hofmann A (2017). Minimal invasive surgical treatment of fragility fractures of the pelvis. Chirurgia (Bucur).

[12] Zhao Y, Zhang S, Sun T (2013). Mechanical comparison between lengthened and short sacroiliac screws in sacral fracture fixation: a finite element analysis. Orthop Traumatol Surg Res.

[13] Heiskanen JT, Kröger H, Pääkkönen M, Parviainen MT, Lamberg-Allardt C, Alhava E (2001). Bone mineral metabolism after total gastrectomy. Bone.

[14] Rino Y, Aoyama T, Atsumi Y, Yamada T, Yukawa N (2022). Metabolic bone disorders after gastrectomy: inevitable or preventable?. Surg Today.

